# User or patient/human or person? Development of a practical framework for applying user-centered design to vulnerable populations for digital transformation in healthcare

**DOI:** 10.1177/20552076251375835

**Published:** 2025-09-08

**Authors:** Angelina Müller, Jannik Schaaf

**Affiliations:** 1 Institute of General Practice, 9173 Goethe University Frankfurt, Frankfurt, Germany; 2 Institute of Medical Informatics, 9173 Goethe University Frankfurt, University Medicine, Frankfurt, Germany

**Keywords:** User-centered design, digital health, health equity, user–computer interface, human factors, vulnerable populations, usability testing

## Abstract

**Objective:**

User-centered design (UCD) is essential in developing healthcare technologies that effectively address user needs, particularly in vulnerable populations. Although UCD traditionally emphasizes direct user involvement, challenges arise when individuals face cognitive, emotional, or structural barriers to participation. In medical settings, integrating UCD with inclusive design principles has been crucial to ensuring equitable healthcare solutions.

**Methods:**

Based on our expertise and a narrative review, we identified challenges and results in UCD approaches in digital health.

**Results:**

The findings reveal key principles for applying UCD in digital health, emphasizing the importance of early user engagement, accessibility, and interdisciplinary collaboration. Studies demonstrate that active involvement of end-users (patients, healthcare providers, and caregivers) through qualitative methods such as interviews, surveys, and usability testing, ideally following iterative design cycles, is critical for designing effective and inclusive digital tools. Accessibility considerations, particularly for vulnerable populations, and integration of digital tools into clinical workflows play an essential role in the process.

**Conclusion:**

To address these challenges, we propose a structured UCD framework for “user–patient–human-friendly” digital transformation in healthcare that guides the development of digital health technologies from initial user research to long-term evaluation. The framework provides a roadmap for designing solutions that are adaptable, inclusive, and sustainable, ensuring that healthcare technologies align with the needs of diverse populations.

## Introduction

User-centered design (UCD), once described by Norman and Draper^1^ and since constantly modified and adapted,^2–5^ plays a crucial role in the development of healthcare technologies. By ensuring that the voices of end-users—such as patients, clinicians, and healthcare providers—are integrated into the design process, UCD promotes the creation of technologies that are more likely to be accepted and effectively used.^1,5,6^ As health systems face growing complexity and increasing reliance on digital tools, incorporating qualitative research methods in UCD has become essential to understanding user needs and barriers.^6,7^ But what happens if the end-users are not able to express their needs or are not aware of them?

In UCD in medical settings, we aim for integration of users, taking cultural contexts into account and following inclusive design principles—an approach commonly used in patient-centered healthcare. Thus, the challenges in vulnerable populations may be similar. For example, it is difficult for ethical reasons to scale the emotional and psychological limitations of a chronically ill person or to compare them with others. In our research, we have conducted various studies applying UCD in several vulnerable populations. These were, for example, patients with rare diseases,^8–10^ patients with chronic diseases,^11^ and human immunodeficiency virus patients.^12,13^ At the same time, we also considered what impact the development of new digital tools could have on the care of vulnerable populations and involved healthcare providers in UCD.^14–17^

There is no doubt that UCD needs to be adapted to a specific scenario,^18–20^ but at the same time, this approach raises the question of how selective applications of the design can enable comparability. In literature, there have been multiple initiatives to address this gap by developing a framework capable of summarizing all guidelines for a successful UCD approach or referring to them for a specific clinical condition or disease.^21–23^ Farao et al. introduced a UCD framework tailored to mHealth, emphasizing the importance of user involvement in all stages of development—from ideation to post-launch evaluation.^24^ This framework guides the design of mHealth apps by focusing on how users interact with these technologies and ensuring that their feedback is actively sought and implemented. Lee and Lee propose that a UCD framework for digital health integrates inclusive design thinking with methodologies from human-centered design, human factors and ergonomics, agile development, and universal design to enhance inclusivity,^25^ emphasizing stakeholder engagement, rigorous evaluation, and digital tool support would improve user acceptance and implementation. Aligning with established models like a five-phase design-thinking framework while ensuring context-aware, inclusive strategies would yield more effective, user-driven solutions. Eventually, Wienert et al. highlight the need for a precise definition of intervention, and differentiation between digital interventions and digital public health interventions.^26^ According to the authors, digital public health interventions should be examined from three perspectives. First, the World Health Organization framework for Essential Public Health Functions^27^ should help define public health activities and interventions. Second, the National Institute for Health and Care Excellence (NICE) Evidence Standards Framework for Digital Health Technologies^28^ should be used to classify digital interventions and set evidence standards. Combining these frameworks allows for categorization based on public health areas and user interaction levels. Lastly, the authors suggest user involvement in development, as proposed by Wright,^29^ as crucial to acceptance. Higher levels of participation can lead to more effective interventions with co-determination (decision-makers consult with the target group, engage in negotiations, and give them a say in decisions) and self-organization (the target group takes full responsibility for the project) being most effective. Prioritizing these higher levels of participation can result in more meaningful and impactful digital public health interventions. In the context of vulnerable populations, which are often associated with digital public health interventions, it should also be considered that in literature we find a liberal use of the terms: user-centered, human-centered, or patient-centered design or approach. This indirectly indicates such aspects would have a similar implication on the design or take similar ethical or social aspects into account. Ideally, especially in vulnerable populations, the terminology of UCD should inherently encompass consideration of an individual's personal background and holistic needs in all their complexity, even when the focus is solely on user behavior.

Therefore, the aim of this paper is to propose a framework for UCD solutions in vulnerable populations, drawing on a range of studies that focus on the application of UCD in implementation of healthcare technologies. By synthesizing findings from these studies, we aim to outline a practical framework that emphasizes the iterative design, involvement of relevant stakeholders, and context-specific adaptations necessary for successful UCD and consequently successful implementation of healthcare technologies.

## Methods

This study was primarily conducted as a narrative review.^30^ Our objective was to synthesize the current knowledge and practical approaches for applying UCD principles in the development of digital health technologies, particularly for vulnerable populations. The narrative review approach was chosen to allow for a comprehensive integration of diverse literature and empirical studies, providing context-specific insights and recommendations for the development of framework.^31^ It is defined as an effort to summarize existing literature without following a strictly systematic approach. In contrast to systematic reviews—which require a clearly defined research question, a structured search strategy, and a comprehensive synthesis of all relevant studies—a narrative review does not adhere to methodological standards, particularly in how the literature is searched and selected.^32^

### Sources of information and search criteria

A targeted literature search was performed across major scientific databases, including Medline, Embase, CINAHL, PsycInfo, and IEEE Xplore. There was no restriction to the search period. The search focused on studies addressing the development, implementation, or evaluation of UCD frameworks or methods in healthcare settings, with attention to chronic conditions and populations facing barriers to healthcare access.

The search strategy combined terms related to “user-centered design,” digital health,” and “vulnerable populations.” Primary research articles and relevant reviews were both considered. Reference lists of key publications were also screened to identify additional studies of interest.

The literature review was conducted by experts in the field. AM is a senior researcher with extensive clinical experience in general practice as well as research in digital health, while JS is a senior researcher specializing in digital health. Both researchers have expertise in applying UCD principles to vulnerable populations.

### Study selection

Studies were included if they described the application of UCD principles in digital health, especially those involving vulnerable or chronically ill populations. Both qualitative and quantitative studies, as well as methodological papers and case studies, were eligible for inclusion. No strict inclusion or exclusion criteria were applied.

### Data extraction and framework development

During the data extraction process, the extraction categories were inductively chosen based on the literature search and analysis of the included studies.

The categories were iteratively refined and adjusted as new insights emerged from subsequent studies, ensuring comprehensive coverage of all relevant framework components.

### Bias reduction

To minimize potential bias throughout the literature review and framework development, we implemented a double screening process and continuous reflection.^33^ All studies were independently screened by the two authors at both the title/abstract and full-text stages, with discrepancies resolved through discussion or, if necessary, consultation with a third researcher.

## Results

### Framework development

The initial search identified 55 articles. After removing one duplicate, 54 articles remained. Screening the titles and abstracts led to the exclusion of 35 articles that did not address vulnerable or chronically ill populations. This process resulted in a final selection of 19 articles (see Supplemental files).

The selected studies exhibit several common principles and methodologies in the application of UCD, which is crucial for the development of digital health interventions. In these studies, UCD principles underscore the importance of a comprehensive understanding of users, their specific needs, and the contexts in which they engage with health-related technologies. A central tenet across all studies is the iterative and participatory nature of the design process, which prioritizes user involvement at every stage of development. A notable commonality across these studies was also the active involvement of users from the outset of the design process. Several studies emphasize the importance of conducting thorough user research, engaging end-users—such as patients, healthcare providers, and caregivers—through interviews, surveys, focus groups, and contextual inquiries. For example, Scheerens et al. supported the involvement of older adults in the design of eHealth tools to ensure that solutions address their unique health challenges and needs.^34^ The study advocates co-designing digital health solutions with the target user community to ensure inclusivity and effectiveness. Levander et al. enrich this aspect by adding a focus on digital health equity, and their case study underlined the importance of involving all stakeholders (patients, healthcare professionals, and policymakers).^18^

An early-stage engagement was considered pivotal in gaining an in-depth understanding of users’ needs, challenges, preferences, and limitations with respect to existing solutions or their absence. The insights gained from this research often lead to the development of personas and use cases, which guide design decisions and ensure the product is aligned with user requirements.^13,14^ Bangash et al. investigated integrating a clinical decision support tool for familial hypercholesterolemia into the workflow of healthcare providers. Physicians identified a number of barriers that were likely to reduce the utilization of the tool in clinical practice,^23^ the most common barrier being the increasing cognitive burden on providers due to implementation of various digital infrastructures and their complexity and limited time during clinical encounters. Addressing such barriers presents considerable challenges; however, these challenges may be mitigated by providing clear and comprehensive explanations of the new tool to all stakeholders, thereby reducing the cognitive load associated with its adoption and use.

An aspect consistently mentioned in the studies was the significance of an iterative design process. UCD frameworks commonly incorporate multiple cycles of prototyping, testing, and refining the design based on user feedback. Many of these studies include usability testing of both low-fidelity and high-fidelity prototypes to assess how real users interact with the system and identify usability issues. Neff et al., Noll et al., and Schaaf et al. included multiple cycles throughout the process.

Another recurring theme in UCD studies is the focus on accessibility and inclusivity. Most studies consciously designed products that can be employed by a broad spectrum of users, including older adults, individuals with disabilities, and users with varying levels of health literacy. Incorporation of accessibility features such as high-contrast modes, voice input, and screen readers was regularly advised, demonstrating a commitment to ensuring equitable access to digital health solutions. Nonetheless, only a minority of studies described specific measures. Usability testing, on the other hand, was performed in all of the prospective studies, regardless of the digital technology aimed at implementation—wearables (Lu et al.),^35^ applications (Reimer et al.), or brain–computer interface applications (Kübler et al.). Most of the studies used validated questionnaires or semi-structured interviews to measure usability.

Finally, the studies highlight the role of collaborative multidisciplinary design, where designers work closely with healthcare professionals, patients, and other stakeholders, not only during the development process but ideally after release or even long term. Lee and Lee, for example, recommend a combination of continuous validation, feedback loops, consortium engagement on digital implementation, and independent maintenance collectively as a strategy for sustaining user participation.^25^

In conclusion, the selected studies collectively affirm that a successful UCD framework for digital health interventions involves comprehensive user research, iterative design and testing, accessibility considerations, and collaborative multidisciplinary efforts. To ensure fairness, equity, and inclusivity in this design, the framework should be maintained in an adaptive and iterative manner. Consequently, it serves as an initial impetus and guiding reference for both the current implementation and future advancements. We have summarized and illustrated the findings in the next section. Recommendations for application in practice are presented as key considerations.

### Practical framework for UCD in digital transformation of healthcare

#### Understanding user needs and context

**Figure 1.**
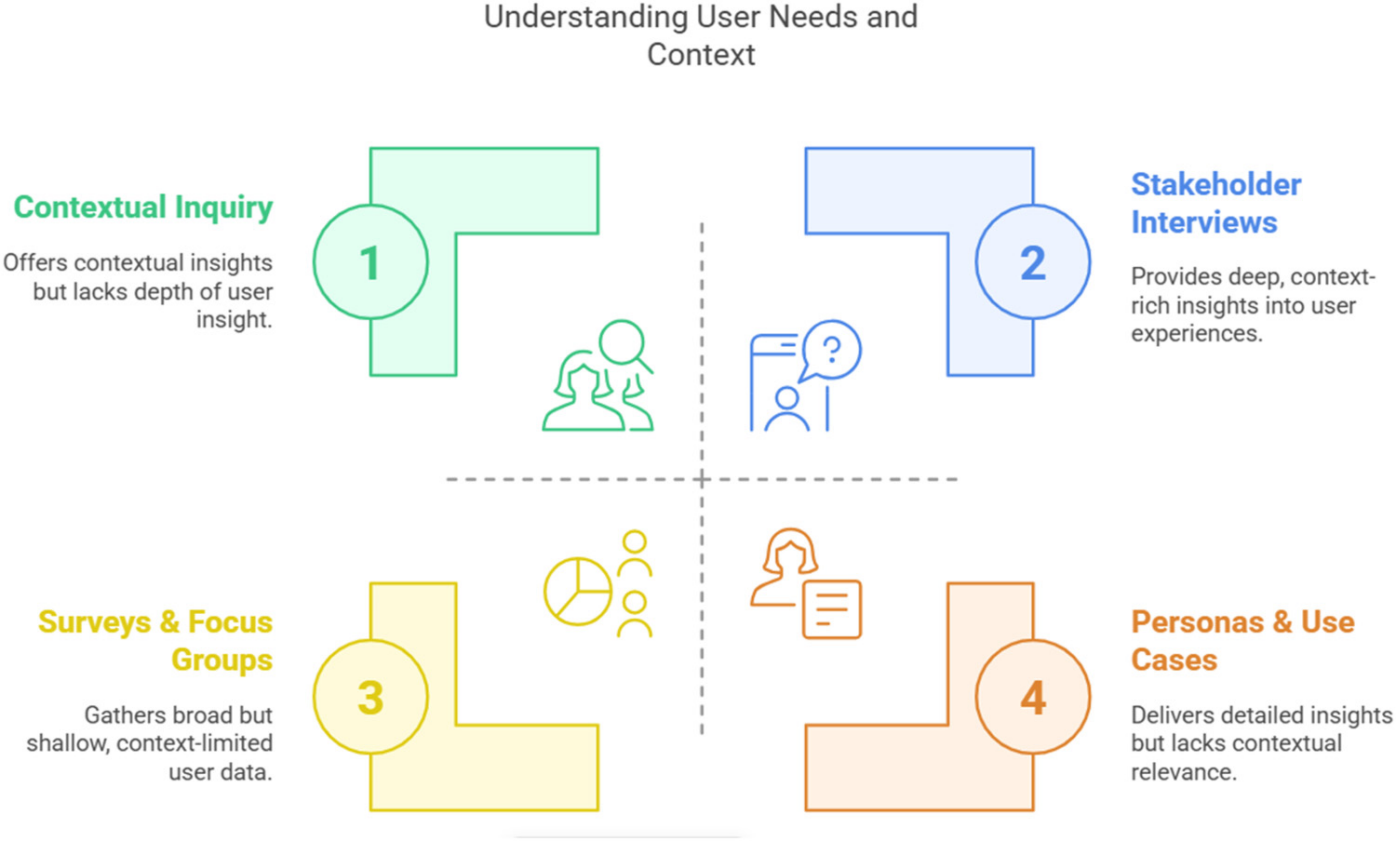
Methods for understanding user needs and context.

The objective is to gather detailed insights into user needs, challenges, and the context in which medical software will be used. Furthermore, especially in vulnerable population groups, creation of a productive yet comfortable and secure working atmosphere is crucial. The activities involved in this phase (as shown in Figure 1) include conducting stakeholder interviews to gather in-depth insights from end-users, such as patients and healthcare providers, to better understand their experiences, preferences, and pain points. Additionally, contextual inquiry is performed by observing users in their natural environments to analyze how they interact with current solutions or the absence thereof. Surveys and focus groups are also utilized to obtain a broader understanding of user preferences, behaviors, and unmet needs. Based on these findings, user personas and specific use cases are developed to guide the design process effectively.

Key considerations during this phase include identifying the primary pain points users encounter in their daily lives, particularly those concerning health-related behaviors. It is also essential to understand barriers to access, technology adoption, and health literacy, especially for vulnerable groups such as elderly individuals or populations with diverse needs or chronic disease(s). Although the term “user” is appropriate in this setting, human factors, personal and social determinants, and their impact on user behavior should be taken into account.

#### Defining requirements and conceptual design

**Figure 2.**
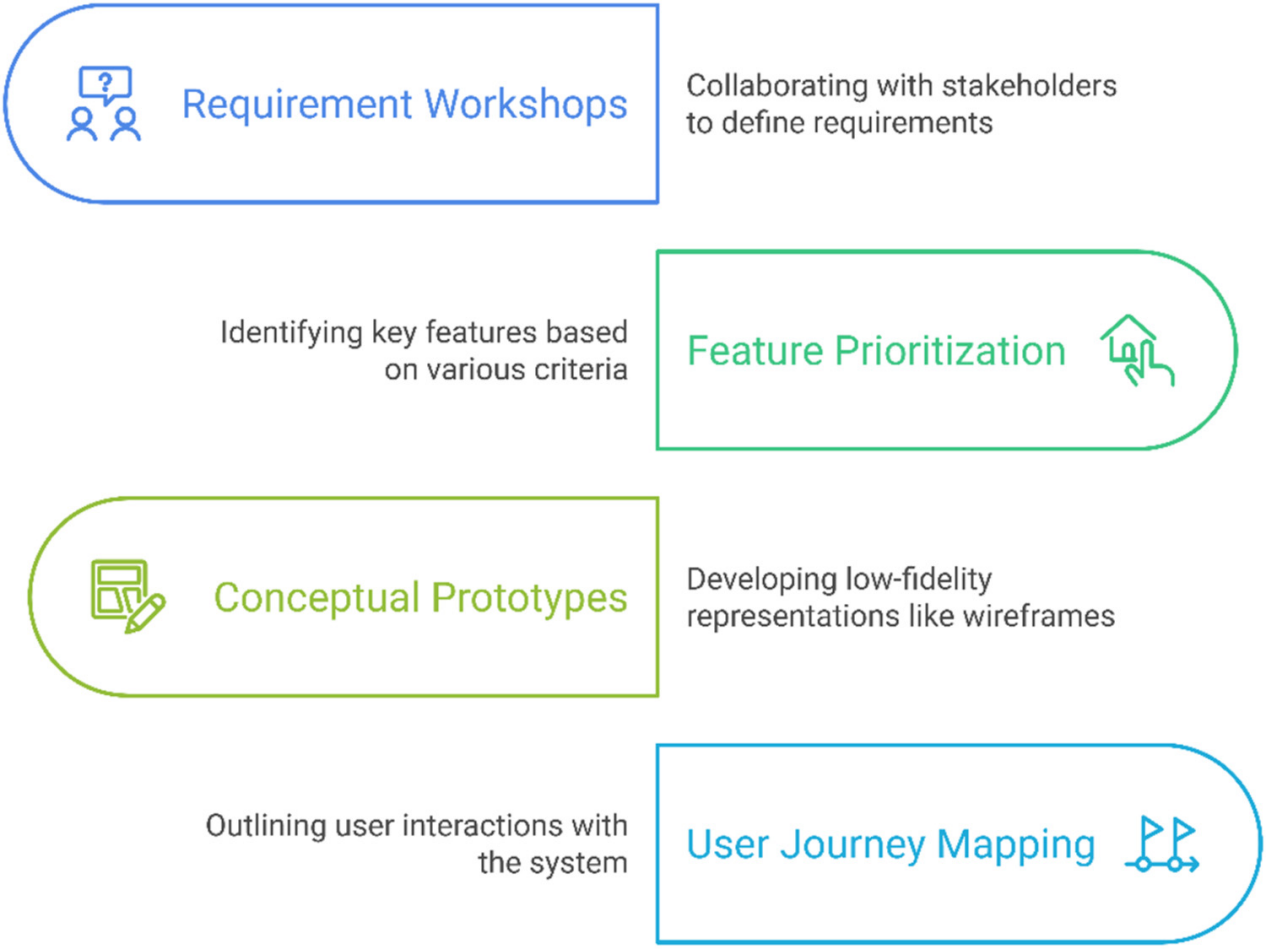
Methods for defining requirements and conceptualizing the design.

The objective is to translate user needs into concrete design requirements and early-stage concepts. The design and development process involves several key activities (see Figure 2) aimed at creating user-centered and effective solutions. First, requirement workshops are conducted to collaborate with users and other stakeholders, such as clinicians, caregivers, and designers. These workshops help define both functional and non-functional requirements, ensuring that the system aligns with the needs of its target audience. Next, feature prioritization is carried out to identify the most important features based on user needs, technical feasibility, and potential health impact. This step ensures that resources are focused on delivering maximum value to users. Following this, conceptual prototypes are developed in the form of low-fidelity representations such as wireframes or mockups. These prototypes serve as visual tools to illustrate the user interface and functionality of the system. Finally, user journey mapping is performed to outline the user's interaction with the system.

Key considerations include ensuring features directly address user pain points and improve user experience. Furthermore, focus on inclusivity and accessibility, ensuring solutions work for a wide range of users with diverse needs (e.g. older adults, individuals with disabilities), is essential.

#### Design and prototyping

**Figure 3.**
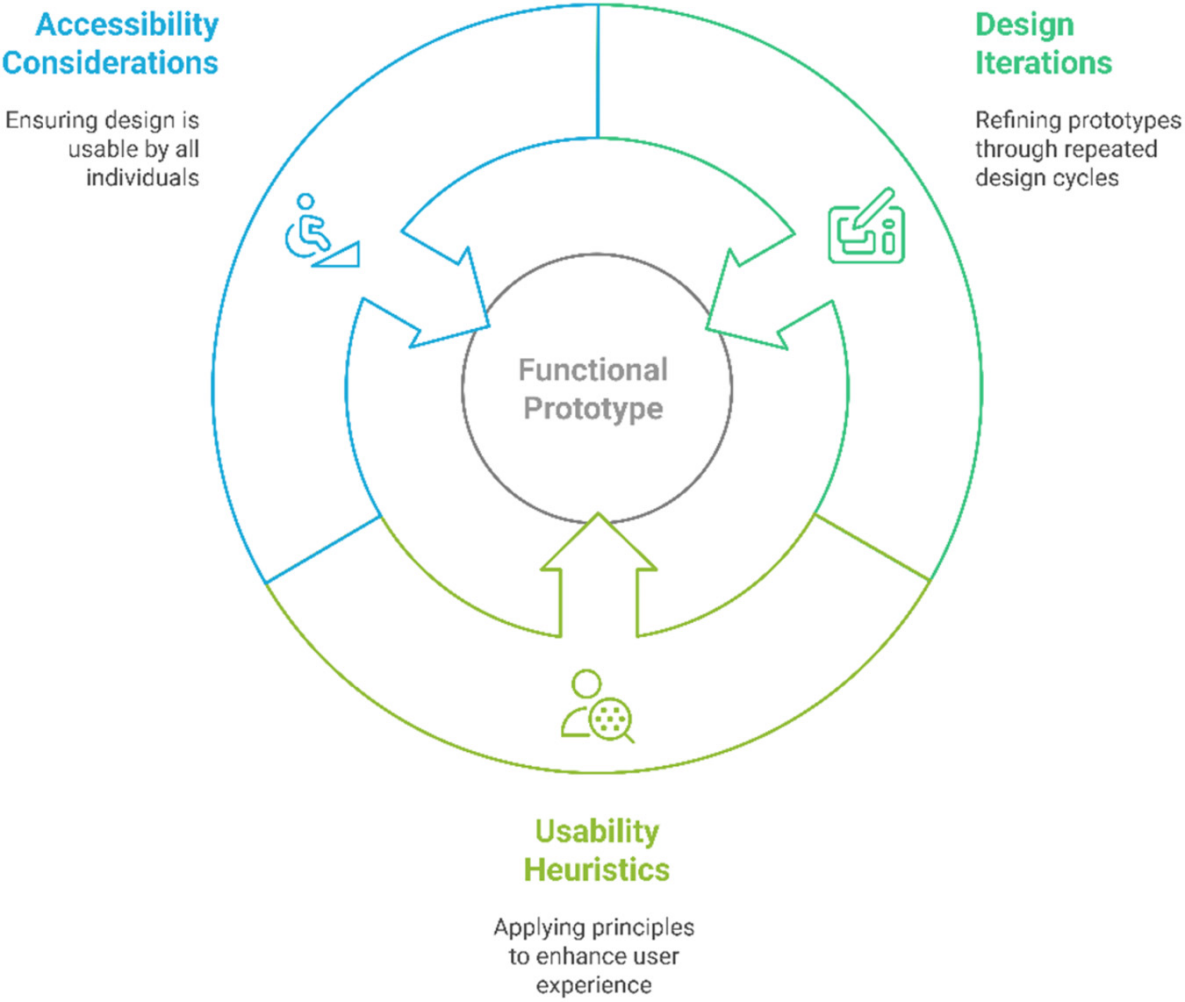
Methods for creating functional prototypes based on the defined requirements and conceptual designs.

The objective is to create functional prototypes based on the defined requirements and conceptual designs. In this phase, several key activities (see Figure 3) are carried out to refine the design and enhance the user experience. One of the primary tasks is conducting design iterations. These prototypes closely resemble the final design and emphasize both visual and interactive elements, ensuring a polished and user-friendly interface. Another crucial aspect is the application of usability heuristics. By adhering to core usability principles such as simplicity, clarity, feedback, and consistency, the design becomes more intuitive and efficient for users. These heuristics help identify and resolve potential usability issues early in the process. Additionally, accessibility considerations play a vital role in making the design inclusive for users with diverse abilities. Features such as voice commands, compatibility with screen readers, and high-contrast modes are incorporated to ensure that the product can be effectively used by individuals with varying needs. By integrating these accessibility measures, the design becomes more user-friendly and compliant with accessibility standards.

Key considerations include ensuring an intuitive and easy-to-navigate design, particularly for users with low digital literacy. Additionally, emphasis is placed on personalization and customization to address users’ specific needs, such as offering adjustable settings for different health conditions.

#### User testing and feedback

**Figure 4.**
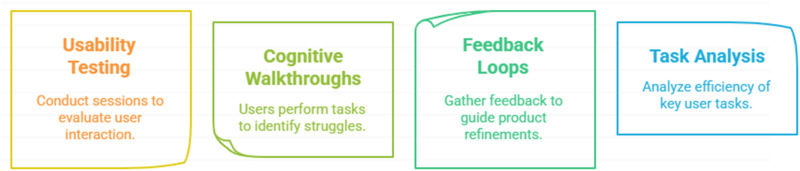
How to perform user testing and receive feedback.

The objective is to test prototypes with end-users to gather feedback and identify issues or areas for improvement. The activities include (see Figure 4) usability testing, where real users interact with the prototype to identify challenges, bottlenecks, and usability issues. Additionally, cognitive walkthroughs are conducted, allowing users to go through specific tasks to uncover areas where they struggle. Iterative feedback loops help gather qualitative and quantitative insights through surveys, interviews, or analytics tools, guiding refinements. Furthermore, task analysis focuses on key user tasks, evaluating how efficiently and effectively they can be completed.

Key considerations include conducting diverse testing that involves different user segments to capture a wide range of perspectives. Additionally, frequent iterations and incremental changes based on feedback are essential to continuously enhance usability.

#### Refinement and iteration

**Figure 5.**
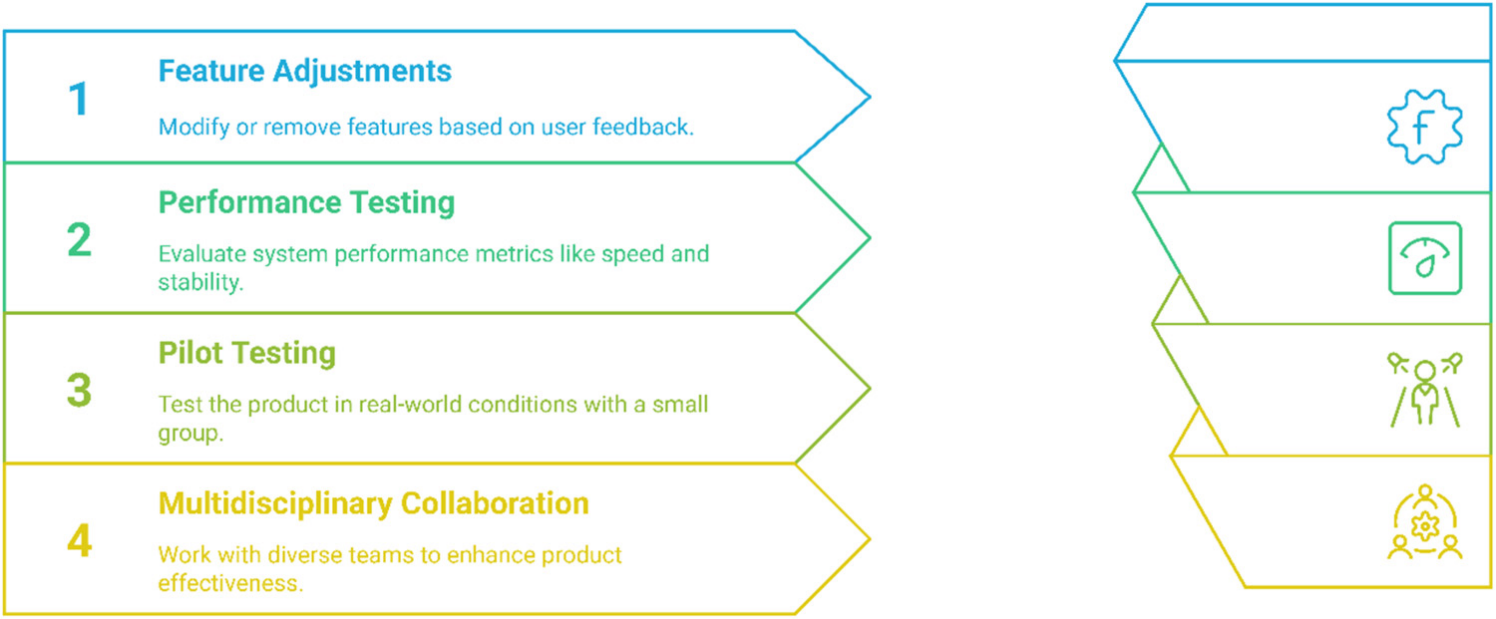
Iterative protocol design.

The objective is to refine the system based on user testing results and prepare it for wider implementation. Feature adjustments involve refining or eliminating features based on user feedback, ensuring that the design accommodates most users while allowing for individual customization (see Figure 5). Performance testing assesses system aspects such as response times, stability, and accessibility. Additionally, pilot testing is conducted with a small group of users in real-world conditions to evaluate the solution's practical effectiveness. Multidisciplinary collaboration plays a crucial role, bringing together developers, healthcare professionals, and end-users to enhance the system's feasibility and overall effectiveness.

Key considerations include ensuring that the final system effectively meets its main goals and user needs. Additionally, ongoing usability evaluation is essential, with a particular focus on long-term use and user engagement.

#### Evaluation and continuous improvement

**Figure 6.**
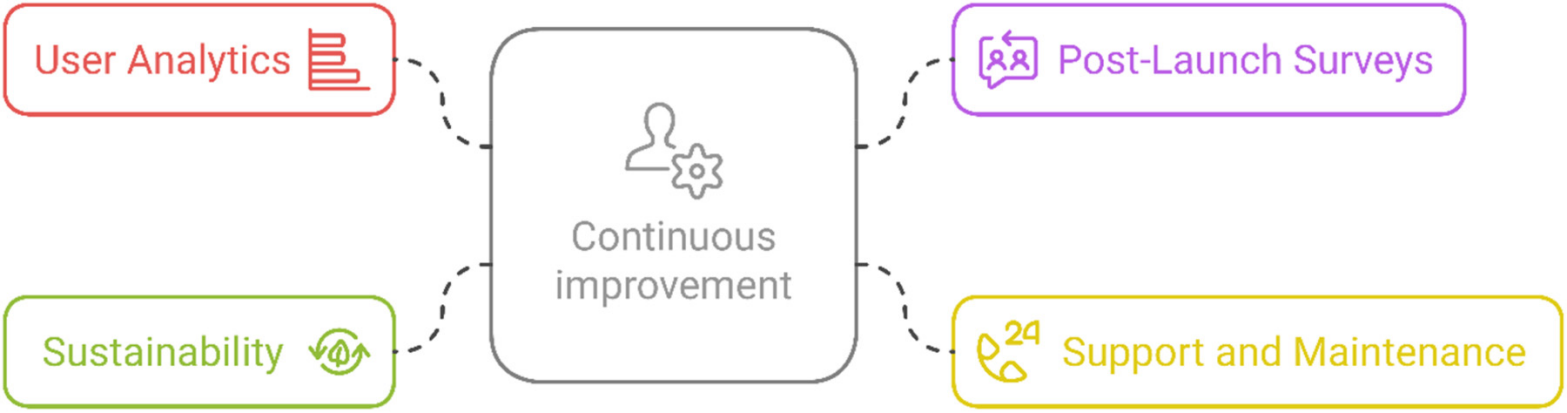
Description of the evaluation approach.

The objective behind a sustainable evaluation is to continuously evaluate the product after launch, incorporating user feedback and adapting to evolving needs (see Figure 6). The activities include user analytics, where data on user engagement is collected and analyzed to identify areas for improvement. Post-launch surveys and interviews are conducted to track user satisfaction and uncover any remaining pain points. Ongoing support and maintenance ensure that users receive troubleshooting assistance, updates, and new features based on feedback. Additionally, sustainability is a key focus, ensuring that the design remains adaptable over time to accommodate new technologies, research, and evolving user needs.

Key considerations include leveraging data analytics to guide decisions on the system's design and functionality. Additionally, user feedback should be continuously incorporated into regular updates to ensure the product evolves in line with users’ needs and preferences.

#### Long-term engagement and behavior change

**Figure 7.**
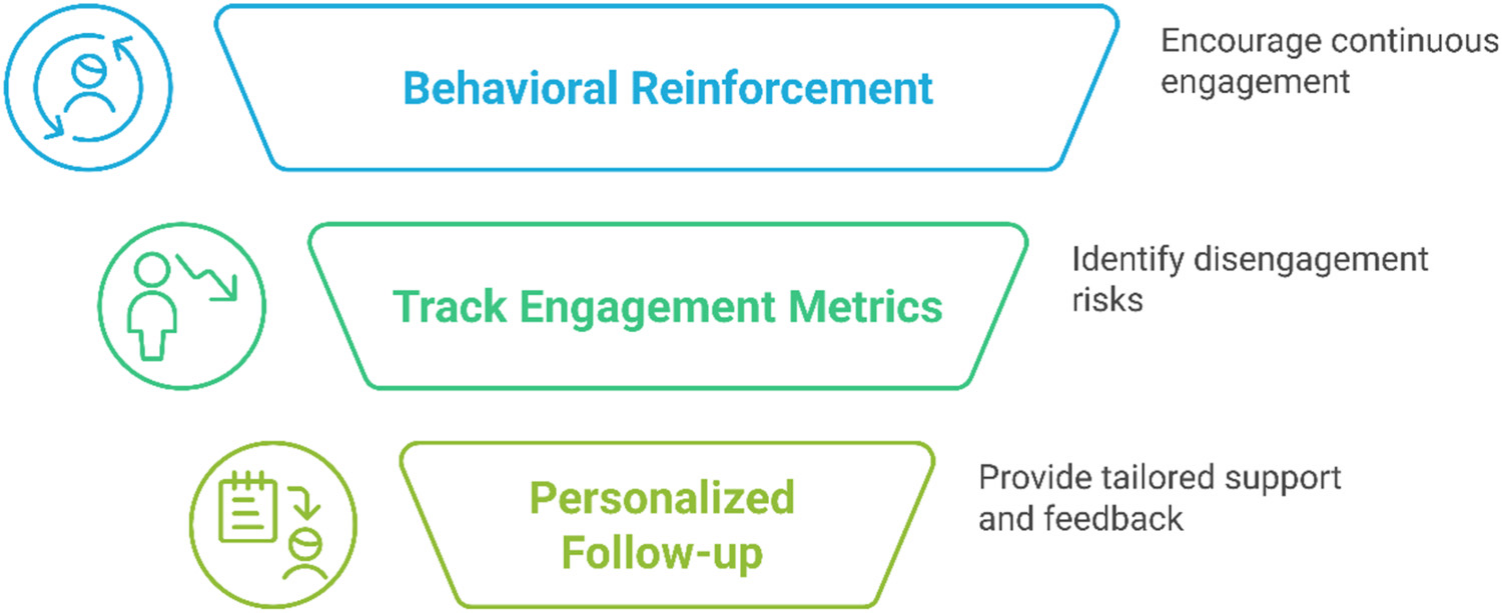
Ideas for a sustainable evaluation approach.

The objective is to ensure that users continue to engage with the technology over the long term and that the intervention is effective in promoting health behavior change. Behavioral reinforcement involves implementing features that promote continuous engagement, such as reminders, motivational feedback, and rewards (see Figure 7). Engagement metrics are tracked to identify users at risk of disengagement, allowing for timely support. Additionally, personalized follow-up provides tailored interventions, support, and feedback based on users’ ongoing behavior and progress.

Key considerations include designing the system to empower users by giving them control over their health data and progress. Additionally, it is important to ensure that the intervention remains flexible and adapts to users’ evolving needs, particularly in long-term health management scenarios.

## Discussion

This paper introduces a framework for UCD solutions in vulnerable population groups, synthesizing insights from various studies on the application of UCD in healthcare technology implementation. The framework provides structured guidance on integrating qualitative research, iterative design processes, and stakeholder involvement to enhance usability, inclusivity, and real-world impact. By building on existing UCD principles and adapting them to the complexities of vulnerable populations, this framework aims to bridge gaps in current methodologies and offers a comprehensive approach to designing digital health interventions. It is based on a narrative literature review, experiences in conducted studies, existing frameworks such as the IDEAS toolkit by Mummah et al.^19^ and a methodology review on design thinking by Riede et al.^36^ aiming to integrate the latest suitable variants.

### Strengths

Among the key strengths of this framework is its emphasis on adapting UCD principles to address the specific challenges faced by vulnerable populations. While traditional UCD approaches prioritize user involvement, this framework highlights the need for deeper engagement strategies when users may struggle to articulate their needs due to health conditions or sociocultural barriers. By incorporating iterative co-design processes and multidisciplinary collaboration, the framework enables more context-sensitive and tailored intervention development.

Another strength lies in its structured approach to stakeholder integration. The framework underscores the importance of engaging healthcare providers alongside patients, ensuring that both perspectives are reflected in the design process. This dual focus helps mitigate barriers such as cognitive overload in clinical settings, a factor frequently cited in studies exploring digital tool adoption among healthcare professionals.

Furthermore, this framework actively promotes accessibility and inclusivity. While many UCD studies acknowledge these aspects, specific measures for ensuring digital equity remain underexplored. By integrating accessibility features like voice commands, high-contrast displays, and simplified interfaces, this framework aligns with best practices for inclusive digital health intervention design. It also highlights the role of usability testing across multiple iterations to refine intervention effectiveness and enhance user experience. Lastly, it aims to sensibilize a complex intervention approach in implementing healthcare technology as proposed in public health.^37^

### Limitations

Despite its contributions, several limitations exist within this framework. Implementing UCD in vulnerable populations poses ethical and methodological challenges, as direct user engagement may be constrained by factors such as cognitive impairments, low health literacy, or socioeconomic limitations. While qualitative methods can provide valuable insights, their application may be hindered by recruitment difficulties or potential biases in self-reported data. A major limitation is also that the definition of vulnerable populations is comprehensive, whereas we were only able to highlight one aspect of it by focusing on healthcare and restrictions due to health conditions. We recommend extending a framework approach in other settings as well. Initializing case studies or pilot studies that incorporate the framework would be favorable.

Additionally, balancing flexibility with comparability remains a challenge. UCD is inherently adaptable, but selectively applying its principles across different contexts raises questions about standardization. This framework does not aim to replace existing UCD models but rather to complement them by offering guidance on addressing unique challenges associated with vulnerable populations.

Finally, while the framework emphasizes stakeholder collaboration, practical difficulties may arise in multidisciplinary teams, including conflicting priorities or resource constraints. Future research should explore strategies for optimizing team dynamics and ensuring sustainable integration of user insights throughout the development lifecycle.

While the proposed framework offers a structured and adaptable approach, several methodical limitations should be considered: the study was conducted as a narrative review rather than a systematic or scoping review. This means that the literature search and selection process did not follow a predefined protocol, potentially introducing selection bias and limiting reproducibility.^38^ It cannot be completely ruled out that thematically appropriate articles have not been considered, especially as the terms used here are not subject to any internally consistent terminology. Without strict inclusion or exclusion criteria, the selection of studies may reflect the authors’ perspectives or areas of expertise, which could influence the objectivity of the findings.

Vulnerable populations often have an increased need for safeguarding their personal health data, which underscores the importance of embedding data protection principles—such as privacy by design and privacy by default—already during the early stages of system development. These principles ensure that privacy is not an afterthought, but a foundational component of any digital health intervention. Moreover, given that vulnerable groups frequently express greater concerns or uncertainty regarding digital technologies and data sharing, a UCD process should actively address issues of trust and data protection from the users’ perspective. This dimension of UCD appears to be underrepresented in the studies reviewed and should be considered more thoroughly in future research and system development.

## Conclusion

In conclusion, UCD frameworks tailored for vulnerable populations in digital health must prioritize deep user engagement, iterative co-design, and multidisciplinary collaboration to address unique challenges and promote inclusivity. Structured stakeholder integration and accessibility measures are essential for ensuring that both patients and healthcare providers’ perspectives shape technology solutions, while usability testing across multiple iterations enhances effectiveness and user experience. Future studies should focus on refining the framework's applicability across diverse healthcare settings and evaluating its effectiveness in real-world implementation.

## Supplemental Material

sj-docx-1-dhj-10.1177_20552076251375835 - Supplemental material for User or patient/human or person? Development of a practical framework for applying user-centered design to vulnerable populations for digital transformation in healthcare Supplemental material, sj-docx-1-dhj-10.1177_20552076251375835 for User or patient/human or person? Development of a practical framework for applying user-centered design to vulnerable populations for digital transformation in healthcare by Angelina Müller and Jannik Schaaf in DIGITAL HEALTH

sj-docx-2-dhj-10.1177_20552076251375835 - Supplemental material for User or patient/human or person? Development of a practical framework for applying user-centered design to vulnerable populations for digital transformation in healthcare Supplemental material, sj-docx-2-dhj-10.1177_20552076251375835 for User or patient/human or person? Development of a practical framework for applying user-centered design to vulnerable populations for digital transformation in healthcare by Angelina Müller and Jannik Schaaf in DIGITAL HEALTH

sj-docx-3-dhj-10.1177_20552076251375835 - Supplemental material for User or patient/human or person? Development of a practical framework for applying user-centered design to vulnerable populations for digital transformation in healthcare Supplemental material, sj-docx-3-dhj-10.1177_20552076251375835 for User or patient/human or person? Development of a practical framework for applying user-centered design to vulnerable populations for digital transformation in healthcare by Angelina Müller and Jannik Schaaf in DIGITAL HEALTH

sj-docx-4-dhj-10.1177_20552076251375835 - Supplemental material for User or patient/human or person? Development of a practical framework for applying user-centered design to vulnerable populations for digital transformation in healthcare Supplemental material, sj-docx-4-dhj-10.1177_20552076251375835 for User or patient/human or person? Development of a practical framework for applying user-centered design to vulnerable populations for digital transformation in healthcare by Angelina Müller and Jannik Schaaf in DIGITAL HEALTH
