# Supplementary material for: User or patient/human or person? Development of a practical framework for applying user-centered design to vulnerable populations for digital transformation in healthcare
Source: Digit Health. 2025 Sep 8;11:20552076251375835. doi: 10.1177/20552076251375835 (PMC12417668; doi:10.1177/20552076251375835)
Supplement: sj-docx-1-dhj-10.1177_20552076251375835 - Supplemental material for User or patient/human or person? Development of a practical framework for applying user-centered design to vulnerable populations for digital transformation in healthcare [file sj-docx-1-dhj-10.1177_20552076251375835.docx]

# Selected studies

Aji, Melissa; Gordon, Christopher; Stratton, Elizabeth; Calvo, Rafael A.; Bartlett, Delwyn; Grunstein, Ronald; Glozier, Nick (2021): Framework for the Design Engineering and Clinical Implementation and Evaluation of mHealth Apps for Sleep Disturbance: Systematic Review. In: *Journal of medical Internet research* 23 (2), e24607. DOI: 10.2196/24607.

Alberts, Nicole M.; Badawy, Sherif M.; Hodges, Jason; Estepp, Jeremie H.; Nwosu, Chinonyelum; Khan, Hamda et al. (2020): Development of the InCharge Health Mobile App to Improve Adherence to Hydroxyurea in Patients With Sickle Cell Disease: User-Centered Design Approach. In: *JMIR mHealth and uHealth* 8 (5), e14884. DOI: 10.2196/14884.

Arsand, Eirik; Demiris, George (2008): User-centered methods for designing patient-centric self-help tools. In: *Informatics for health & social care* 33 (3), S. 158–169. DOI: 10.1080/17538150802457562.

Bangash, Hana; Pencille, Laurie; Gundelach, Justin H.; Makkawy, Ahmed; Sutton, Joseph; Makkawy, Lenae et al. (2020): An Implementation Science Framework to Develop a Clinical Decision Support Tool for Familial Hypercholesterolemia. In: *Journal of personalized medicine* 10 (3). DOI: 10.3390/jpm10030067.

Ghaben, Suad J.; Mat Ludin, Arimi Fitri; Mohamad Ali, Nazlena; Beng Gan, Kok; Singh, Devinder Kaur Ajit (2023): A framework for design and usability testing of telerehabilitation system for adults with chronic diseases: A panoramic scoping review. In: *Digital health* 9, 20552076231191014. DOI: 10.1177/20552076231191014.

Helmark, Charlotte; Ahm, Robert; Brandes, Axel; Skovbakke, Søren J.; Nielsen, Jens Cosedis; Frostholm, Lisbeth et al. (2025): Development of an online psychological intervention to prevent depression in patients with atrial fibrillation: lessons learned in a user-centered design study. In: *Pilot and feasibility studies* 11 (1), S. 2. DOI: 10.1186/s40814-024-01586-1.

Kübler, Andrea; Holz, Elisa M.; Riccio, Angela; Zickler, Claudia; Kaufmann, Tobias; Kleih, Sonja C. et al. (2014): The user-centered design as novel perspective for evaluating the usability of BCI-controlled applications. In: *PloS one* 9 (12), e112392. DOI: 10.1371/journal.pone.0112392.

Levander, Ximena A.; VanDerSchaaf, Hans; Barragán, Vanessa Guerrero; Choxi, Hetal; Hoffman, Amber; Morgan, Emily et al. (2024): The Role of Human-Centered Design in Healthcare Innovation: a Digital Health Equity Case Study. In: *Journal of general internal medicine* 39 (4), S. 690–695. DOI: 10.1007/s11606-023-08500-0.

Lu, Jessica K.; Wang, Weilan; Goh, Jorming; Maier, Andrea B. (2024): A practical guide for selecting continuous monitoring wearable devices for community-dwelling adults. In: *Heliyon* 10 (13), e33488. DOI: 10.1016/j.heliyon.2024.e33488.

Noll, Richard; Voigt, Alexander; Koehler, Susanne; Mueller, Angelina; Stephan, Christoph; Carney, Jonathan et al. (2023): Enhancing HIV Patient Support Through Telehealth: Exploring Design Solutions. In: *Studies in health technology and informatics* 309, S. 150–154. DOI: 10.3233/SHTI230764.

Reimer, Lara Marie; Nissen, Leon; Scheidt, Moritz von; Perl, Benedikt; Wiehler, Jens; Najem, Sinann Al et al. (2024): User-centered development of an mHealth app for cardiovascular prevention. In: *Digital health* 10, 20552076241249269. DOI: 10.1177/20552076241249269.

Schaaf, Jannik; Khouri, Andreas; Zerr, Thomas; Scheidt, Jörg; Neff, Michaela; Storf, Holger (2024): Rare Diseases in Citizen Science - Preliminary Experiences in Developing a Personal Health App. In: *Studies in health technology and informatics* 310, S. 1151–1155. DOI: 10.3233/SHTI231145.

Schaaf, Jannik; Prokosch, Hans-Ulrich; Boeker, Martin; Schaefer, Johanna; Vasseur, Jessica; Storf, Holger; Sedlmayr, Martin (2020): Interviews with experts in rare diseases for the development of clinical decision support system software - a qualitative study. In: *BMC medical informatics and decision making* 20 (1), S. 230. DOI: 10.1186/s12911-020-01254-3.

Schaaf, Jannik; Sedlmayr, Martin; Sedlmayr, Brita; Prokosch, Hans-Ulrich; Storf, Holger (2021): Evaluation of a clinical decision support system for rare diseases: a qualitative study. In: *BMC medical informatics and decision making* 21 (1), S. 65. DOI: 10.1186/s12911-021-01435-8.

Schaaf, Jannik; Sedlmayr, Martin; Sedlmayr, Brita; Storf, Holger (2022): User-Centred Development of a Diagnosis Support System for Rare Diseases. In: *Studies in health technology and informatics* 293, S. 11–18. DOI: 10.3233/SHTI220341.

Schaaf, Jannik; Weber, Timm; Wagner, Michael von; Stephan, Christoph; Carney, Jonathan; Köhler, Susanne Maria et al. (2023): Interviews with HIV Experts for Development of a Mobile Health Application in HIV Care-A Qualitative Study. In: *Healthcare (Basel, Switzerland)* 11 (15). DOI: 10.3390/healthcare11152180.

Schaaf, Jannik; Weber, Timm; Wagner, Michael von; Stephan, Christoph; Köhler, Susanne Maria; Voigt, Alexander et al. (2024): Exploring patient-centered design solutions of a telehealth app for HIV - A qualitative study. In: *International journal of medical informatics* 189, S. 105524. DOI: 10.1016/j.ijmedinf.2024.105524.

Scheerens, Charlotte; Gilissen, Joni; Volow, Aiesha M.; Powell, Jana L.; Ferguson, Clarissa M.; Farrell, David et al. (2021): Developing eHealth tools for diverse older adults: Lessons learned from the PREPARE for Your Care Program. In: *Journal of the American Geriatrics Society* 69 (10), S. 2939–2949. DOI: 10.1111/jgs.17284.

Stuij, Sebastiaan M.; Drossaert, Constance H. C.; Labrie, Nanon H. M.; Hulsman, Robert L.; Kersten, Marie José; van Dulmen, Sandra; Smets, Ellen M. A. (2020): Developing a digital training tool to support oncologists in the skill of information-provision: a user centred approach. In: *BMC medical education* 20 (1), S. 135. DOI: 10.1186/s12909-020-1985-0.

Vannelli, Sara; Visintin, Filippo; Dosi, Clio; Fiorini, Laura; Rovini, Erika; Cavallo, Filippo (2024): A Framework for the Human-Centered Design of Service Processes Enabled by Medical Devices: A Case Study of Wearable Devices for Parkinson’s Disease. In: *International journal of environmental research and public health* 21 (10). DOI: 10.3390/ijerph21101367.
